# An Optimization Method for the Station Layout of a Microseismic Monitoring System in Underground Mine Engineering

**DOI:** 10.3390/s22134775

**Published:** 2022-06-24

**Authors:** Zilong Zhou, Congcong Zhao, Yinghua Huang

**Affiliations:** 1 School of Resources and Safety Engineering, Central South University, Changsha 410083, China; zlzhou@csu.edu.cn (Z.Z.); 215502042@csu.edu.cn (Y.H.); 2 State Key Laboratory of Safety Technology of Metal Mines, Changsha Institute of Mining Research Co., Ltd., Changsha 410012, China

**Keywords:** underground mine, microseismic monitoring, network layout, method optimization, S-V-E-R-V model, monitoring efficiency

## Abstract

The layout of microseismic monitoring (MSM) station networks is very important to ensure the effectiveness of source location inversion; however, it is difficult to meet the complexity and mobility requirements of the technology in this new era. This paper proposes a network optimization method based on the geometric parameters of the proposed sensor-point database. First, according to the monitoring requirements and mine-working conditions, the overall proposed point database and model are built. Second, through the developed model, the proposed coverage area, envelope volume, effective coverage radius, and minimum energy level induction value are comprehensively calculated, and the evaluation reference index is constructed. Third, the effective maximum envelope volume is determined by taking the analyzed limit of monitoring induction energy level as the limit. Finally, the optimal design method is identified and applied to provide a sensor station layout network with the maximum energy efficiency. The method, defined as the S-V-E-R-V model, is verified by a comparison with the existing layout scheme and numerical simulation. The results show that the optimization method has strong practicability and efficiency, compared with the mine’s layout following the current method. Simulation experiments show that the optimization effect of this method meets the mine’s engineering requirements for the variability, intelligence, and high efficiency of the microseismic monitoring station network layout, and satisfies the needs of event identification and location dependent on the station network.

## 1. Introduction

Microseismic monitoring (MSM) represents an important real-time, online, and remote monitoring method that is widely used in many fields, including underground tunnel construction, deep mining, water conservancy, and hydropower projects [1,2,3,4,5,6,7,8,9]. Particularly in the case of underground mine excavation and mining construction processes, the scientific framework and practical layout of MSM station networks are the basis for data acquisition and the promotion of field data interpretation and application [10,11,12]. Therefore, the development of an optimization method for the station layout of an MSM system for underground works and mine engineering is very worthwhile [13]. The integrity and accuracy of monitoring data depend on the effectiveness of the system, and the layout of the MSM network is the primary and core element when constructing an effective MSM system. There are five reasons for this: (1) The measurement accuracy of the source parameters plays a key role in the processing and analysis of MSM data and even disaster prevention; (2) The purpose of the network layout is to determine the closest proven location of the event and the real earthquake occurrence time; (3) The travel time and location are ascertained using data from the established stations; the key parameters generated by the network foundation are subject to the deployment of the network; (4) The layout of the network is a prerequisite and can be optimized with human intervention to the greatest extent in the primary stage; (5) In the context of the state of the art, with the help of big data technology and software programming technology, it is the general trend to conduct a large number of numerical experiments on the indoor network layout scheme, as it has extremely high test repeatability and promotion feasibility. Therefore, studying methods to optimize the network layout of an MSM system is of great significance. 

To date, many methods have been proposed to optimize the layout of MSM networks, mainly falling into two categories: (I) basic mathematical and physics theories and algorithms, and (II) methods combining a theoretical basis and a consideration of engineering practices, improved algorithms, and engineering case application analysis. The advantages and disadvantages of these two approaches are briefly introduced in Table 1 below.

The above two categories of methods are affected by three main limiting factors that lead to substantial efficiency errors in the sensor layout methods in the existing MSM network. Therefore, a processing method that adapts to the changes of times and the needs of the engineering site is urgently needed, to maximize the layout and optimization method for the MSM sensor network.

In recent years, considerable results have been achieved in the field of grid layout optimization methods under the conditions of specific structures in mining engineering sites and other applications [31,32,33]. Kijko et al. [16,17,19] analyzed the network according to the epicenter location and time error model and introduced a study of the residual relationship between the P-wave and S-wave in the average geological model, summing up classical D-value optimization. Ge et al. [25,26] carried out a detailed theoretical analysis of the geometry, principles, layout, and accuracy of the average geological model in their doctoral dissertations, and carried out program development and application in actual working conditions in the later stage. Gong and Dou et al. [27] developed a comprehensive index method based on the D-value optimization theory, formulated the general principles for determining station candidate points and monitoring areas, established the objective function for optimizing the layout of the station network, and proposed a model data preparation module, a genetic algorithm solution module, and a network layout solution positioning the capability evaluation module as the microseismic network layout solution model. These methods are classic and enable more realistic results, but they cannot meet the needs of the current times. This inability is mainly manifested in the grid layout design method that is adopted by most mines, which does not have the flexibility of a rapid response time and cannot adapt to the continuous deepening and dynamic changes in production rhythm and transition efficiency. The combination planning of the grid layout scheme involves a huge number of calculations, and the velocity field and mathematical variables under certain grid layout conditions are complex and changeable. This makes it difficult for a particular algorithm to maintain lasting superiority. Therefore, we propose a fast, efficient, and intelligent network layout method: an MSM network layout method with superior dynamic adaptability that can adapt to the engineering design and field implementation stages and achieve a reliable and sustainable monitoring effect.

**Figure 1:**
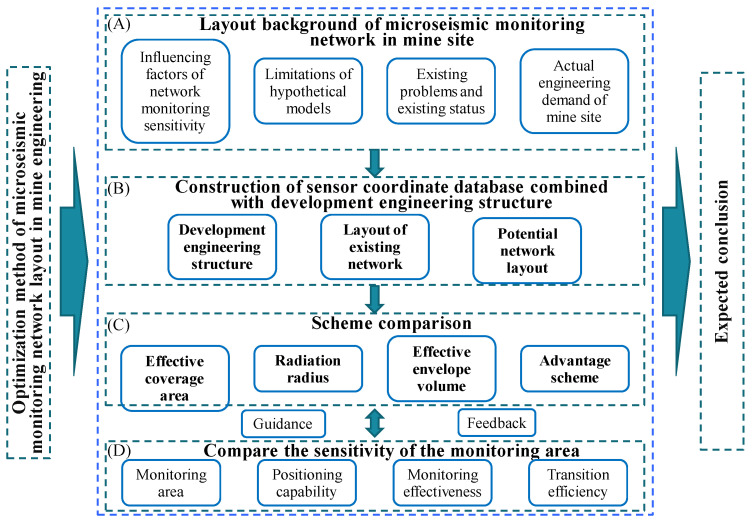
Technical route: (**A**) objective factors; (**B**) subjective factors; (**C**) the main research indicators of this study; (**D**) inspection index.

To further improve the efficiency of the network layout, an optimization method for the station layout of an MSM system for underground mine engineering is proposed, based on the comprehensive calculation and evaluation of the coverage area, envelope volume, effective coverage radius, and minimum energy level induction value of any network. This method firstly constructs an indexed dataset (S-V-E-R-V) from potential sensor placement points, calculates the solutions in the proposed library according to the index values, then weighs and normalizes them and, finally, obtains coverage within the same target range. The results of the maximum efficiency of the network layout plan verify its effectiveness and practicability. The specific innovative technology route is shown in Figure 1 below. 

The outline of this paper is as follows. In Section 2, we introduce the materials and methods in the context of the Xinjiang Ashele Copper Mine, which provides the research base. In addition, we propose a new method in Section 3, including parameters and their definitions, calculation procedures, and formula optimization. Several numerical simulations and case studies are presented in Section 4, wherein the current network layout plan of the mine is analyzed. At the same time, based on our new method, the simulation solution is comprehensively established, and the comparison results of the optimum layout scheme are given. Finally, our main conclusions are summarized in Section 5.

## 2. Materials and Methods

**Figure 2:**
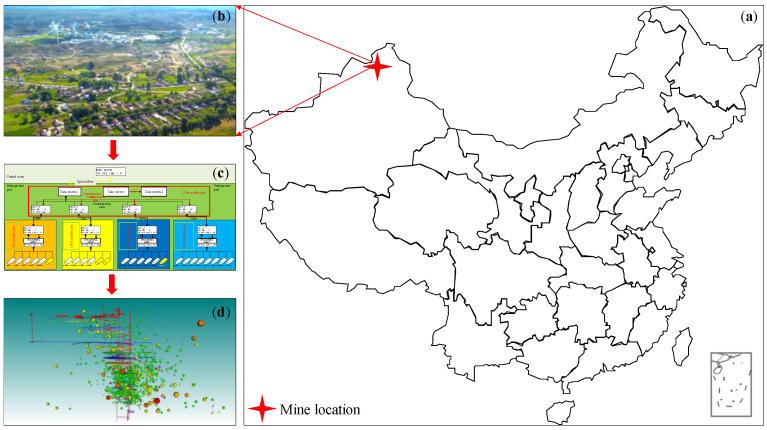
Microseismic monitoring (MSM) system of the Ashele Copper Mine: (**a**) the geographical location of the analysis area; (**b**) geography of the target mine; (**c**) topology map of the microseismic monitoring system of the target mine; (**d**) deployment and monitoring of the microseismic monitoring system.

This paper takes the Xinjiang Ashele Copper Mine as the engineering backdrop. Its mining depth is successively from 502 m in the first phase to 910 m in the second phase, then down to 1243 m in the planned third phase. The MSM service system was fully and effectively applied in the first and second phases. Figure 2 shows the engineering backdrop to this research, including the specific natural geographical location and the topology of the MSM system, particularly the current status of the existing network layout. The aim of this paper is to study and analyze the network layout of the MSM system in the second phase of the project and to optimize and improve the monitoring efficiency of the current network. On this basis, the method in this paper is proposed to optimize the design of the grid layout of the upcoming three-phase project. 

**Figure 3:**
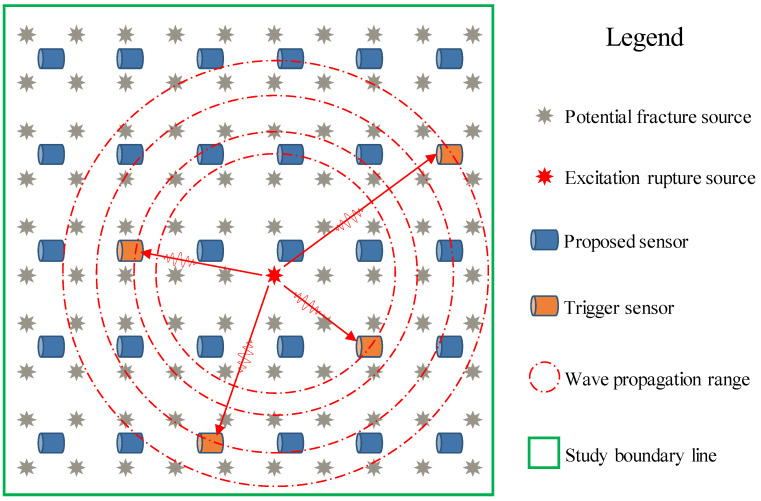
Schematic diagram of monitoring efficiency analysis of the MSM system network layout.

With the increase in the use of MSM systems in mines and other engineering fields, the layout of the network in the early stage of a scheme design is very important. In terms of theoretical research, the theoretical assumptions and data input factors of traditional analysis and optimization methods are dominant, and the feasibility of procedural processing is extremely low, resulting in traditional methods that are time-consuming and labor-intensive and that rely more on the experience of field engineers. To improve the rapid dynamic response of the network layout plan, based on a certain number of sensors, researchers focus on maximizing the entire monitoring range of the sensor envelope and improving the monitoring efficiency. At present, without considering the positioning accuracy and rock mass structure parameters, only a limited number of sensors have the largest coverage area and the largest envelope volume; there are few related studies aimed at improving the monitoring efficiency. A schematic diagram showing the monitoring efficiency analysis of the MSM system network layout is shown in Figure 3. It depicts the physical representation and relationship between potential sensor placement and monitoring energy efficiency.

### 2.1. Principles of Classical Methods and Optimization Basis

The advantages and disadvantages of the microseismic network layout plan are the main factors that determine the performance of MSM, so it is necessary to study the optimal layout of the microseismic network. Regarding the grid layout optimization method, the internationally developed and mature theoretical foundations of the method are the D-value optimization and C-value optimization of Kijko and Mendecki, respectively, in which the objective mathematical function for the evaluation of the grid layout scheme is constructed [19]. The core idea is to minimize the transformation matrix ellipsoid, formed by the occurrence time and coordinate position of multiple unknown source events and other multivariate variables, so as to achieve the optimal network layout effect. Among them, the multivariate variables can enable the comprehensive monitoring performance of a specific network, including microseismic event energy, monitorable radius, the magnitude of the monitored event, the distribution area and probability of major events, and the sensitivity to hypocenter events. 

Based on the uncertainty of different paths and wave speeds, the time parameter is a relatively accurate measurement value; a time-based approximate equation is constructed to obtain an approximate analytical solution:

(1) $$t_{i}=t_{0}+T\left(h,s_{i}\right)+\varepsilon_{i}\;,$$

where $t_{i}$ is the arrival time of the *i*-th triggered sensor to monitor the hypocenter; $t_{0}$ represents the time when the hypocenter occurs; $T\left(h,s_{i}\right)$ denotes the travel time of the wave; and $\varepsilon_{i}$ stands for the travel time error, where $h\left(x_{0},y_{0},z_{0}\right)$ and $s_{i}\left(x_{i},y_{i},z_{i}\right)$ are the source coordinates and the spatial coordinates of the *i*-th triggered sensor, respectively. 

If the most genuine arrival time is required, it is necessary to ensure that the error term in Equation (1) is the smallest, after which the equations regarding the source parameters and travel time parameters can be constructed, as follows:

(2) $$\phi \left(x\right)=\sum^{\;}{\left|t_{i}-t_{0}-T\left(h,s_{i}\right)\right|}^{p}\;$$

where x represents the collection of time and space coordinates of the source $\left(t_{0},x_{0},y_{0},z_{0}\right)$, and where *p* is usually 2.

In a mathematical relationship, if a certain quantity is required to be solved, another predetermined quantity of related eigenvalues is required to be matched and solved. Then, a covariance matrix, considering random error and source detection, can be constructed for the source event x, set at $\left[t_{0},x_{0},y_{0},z_{0};\ldots ;t_{i},x_{i},y_{i},z_{i}\right]$ in Equation (2); the minimization result takes precedence. To minimize the travel-time error, the following mathematical equation is constructed:

(3) $$minimum\;f\left(C_{x}\right)\;\;,\;\;s\in \varphi$$

where $C_{x}$ is the covariance matrix of x; the constants and variables of the f function depend on the physical properties and meaning of the problem under consideration; s represents the set of station coordinates in the network; and φ denotes the spatial domain of the possible station locations.

Based on the optimization theory of the D-value, an approximate confidence ellipsoid of the x parameter is constructed. The estimated value $\hat{x}$ of x and x after the transformation has a specific relationship with the covariance of the x matrix, and it is subject to a certain constant c to keep it stable or convergent. Mathematical deformation and matrix transposition are performed on $C_{x}$ to form the following expression relationship:

(4) $$\left(x-\hat{x}\right)C_{x}^{-1}{\left(x-\hat{x}\right)}^{T}\leq c\;,\;\;c\;\mathrm{is}\;a\;\mathrm{constant}.$$

At this point, the characteristics of the determinant can be used to simplify the solution of the covariance matrix. Specifically, when the propagation process of the randomly occurring microseismic event is described by a complex nonlinear equation, it is not appropriate to directly apply the D-value optimization principle, but it can be used at the initial point. Partial derivative matrices are computed for the common parameters, thereby eliminating computational complexity. 

Assuming that source parameter x can be estimated by any norm p, this results in $C_{x}\propto {\left[A^{T}\;A\right]}^{-1}$, where A is the partial derivative matrix of the calculated arrival time for x, transformed as follows in Equation (5):

(5) $$A=\left(\begin{array}{c} 1,\\ 1, \end{array}\;\begin{array}{ccc} \frac{\partial T_{1}}{\partial x_{0}}, & \frac{\partial T_{1}}{\partial y_{0}}, & \frac{\partial T_{1}}{\partial xz_{0}}\\ \vdots & \vdots & \vdots \\ \frac{\partial T_{n}}{\partial x_{0}}, & \frac{\partial T_{n}}{\partial y_{0}}, & \frac{\partial T_{n}}{\partial xz_{0}} \end{array}\right)\;.$$

According to the matrix characteristics and D-value optimization criteria, in $C_{x}={\left[\det C_{x}^{-1}\right]}^{-1}$, if $\det C_{x}^{\;}$ is to be minimized, then $\det A^{T}A$ can be maximized. 

At this point, if the following Equation (6) can be satisfied, the optimal solution can be obtained:

(6) $$C_{min}=a+b\times \int_{x_{0}}^{x_{i}}f\left(C_{x}\right)dx\;$$

where $C_{min}$ is the analytical solution of minimizing the covariance matrix when other main parameters are added; $C_{x}$ represents the covariance matrix of x; a denotes the constant term of the additional main parameters; b is the correction coefficient term of the additional main parameters and $x_{0}$ is the initial value of the parameter; $x_{i}$ represents the end value of the source parameter; and dx is the integral of x.

In the D-value optimization criterion, in addition to the core key parameters of $C_{x}$, there are still many other hidden unknown parameters or their subset factors that affect the monitoring efficiency of the network layout. In the D-value optimization criterion, the confidence ellipsoid is proportional to $C_{x}$. Combining the special factors and other natural characteristics studied by other scholars, this study conducts the basic and necessary analyses and research based on this core key parameter: considering the target area and attribute function of the network layout monitoring, the envelope formed by the sensor station network space needs to have certain geometric properties. This paper focuses on the comprehensive influence of these basic properties, such as envelope volume, coverage area, radiation radius, and induced energy, to optimize the network layout. 

### 2.2. New Indicators for the S-V-E-R-V Model

**Figure 4:**
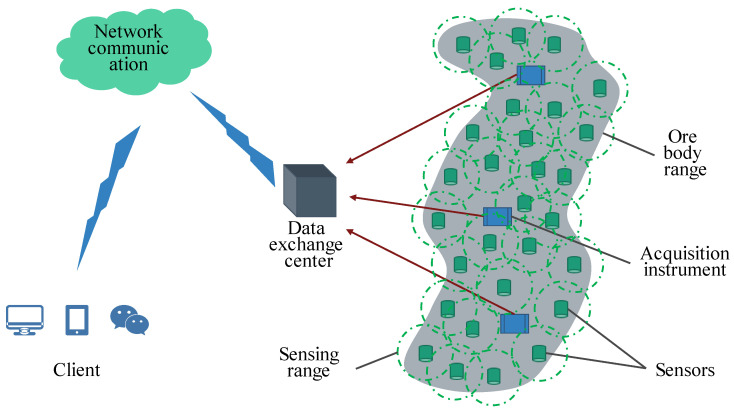
Schematic diagram of the optimization model concept.

Based on the above theory and our analysis of *D*-value optimization and *C*-value optimization, in the process of formulating a covariance data solution for *D*-value optimization, the key parameters of the target are introduced, to form the optimized S-V-E-R-V parameter model; thus, this study’s systematic optimization method and scheme are formed. When arranging the spatial points of sensor stations at mine sites, the site conditions and the adaptability of structures are often considered, while the principles and mechanisms of the network layout are rarely applied. Based on the *D*-value optimization theory, the coverage area of the field network (S), the network envelope volume (V), the limit magnitude energy coverage radius of the network ($R_{E}$), and the effective magnitude sensitivity monitoring envelope volume ($V_{E}$) are primarily considered. When the target monitoring area and spatial scope of the mine are fixed, for a certain epicenter event, the monitoring efficiency of different network layout schemes varies. First, to ensure that the network layout meets the necessary space-monitoring conditions, it is necessary to achieve maximum efficiency in the physical space; second, it is necessary to attain monitoring efficiency regarding the physical magnitude of the event and its detectable radius scale; third, the minimum magnitude sensitivity of the event can be monitored. The volume within the effective space range is used as a guiding parameter for the closed-loop optimization of the model, as shown in Figure 4. This demonstrates the range of monitoring efficacy resulting from actual sensor placement in the model that acts as a basis for the calculations in this study.

#### 2.2.1. S + V

When a source rupture event occurs inside the envelope, the precision and accuracy of localization is much better than when the event occurs outside the envelope. Specifically, when the sensor array within the monitoring target area is established, its three-view projection area is given, and the coverage area is expressed as:

(7) $$S=S_{Pxoy}+S_{Pxoz}+S_{Pyoz}\;,$$

where S is the sum of the projected areas of the three views of the sensor array in the spatial area of the monitoring target range; $S_{Pxoy}$ represents the projected area of the XOY plane in the Cartesian coordinate system; $S_{Pxoz}$ denotes the projected area of the XOZ plane in the Cartesian coordinate system; and $S_{Pyoz}$ stands for the projected area of the YOZ plane in the Cartesian coordinate system.

Specifically, when the sensor array within the monitoring target area is given, and the volume of the space that encloses it is given, the envelope volume is expressed as:

(8) $$V=\int_{z_{min}}^{z_{max}}s_{xoy}dz\;,$$

where *V* is the volume of the space domain that can be enveloped by the sensor array that monitors the spatial area of the target range, and $s_{xoy}$ represents each xoy of the body of the space formed by the sensor array in the Cartesian coordinate system, differentiated in the plumb direction. In the surface geometric area, dz is the differential in the *z* direction, is the lower limit of the geometric space elevation formed by the sensor array, and $z_{max}$ is the upper limit of the geometric space elevation formed by the sensor array.

This is convenient for the comprehensive evaluation of the subsequent overall model. At this point, the geometric space parameters of the sensor array are subjected to the normalization processing and preliminary weighting integration processing at fixed intervals. The normalization of the coverage area is as follows:

(9) $$S_{new}=\frac{S-S_{min}}{S_{max}-S_{min}}\;,$$

where $S_{new}$ is the dimensionless coverage area that is normalized; S represents the sum of the triple-view projected areas of each grid layout scheme; $S_{min}$ denotes the projected area and the minimum value; and stands for the projected area and the maximum value.

The normalization of the envelope volume is handled as follows:

(10) $$V_{new}=\frac{V-V_{min}}{V_{max}-V_{min}}\;,$$

where $V_{new}$ represents the dimensionless envelope volume that is normalized; V denotes the three-dimensional space envelope volume of each grid layout scheme; $V_{min}$ is the minimum envelope volume; and $V_{max}$ stands for the maximum envelope volume.

Assuming that the weight ratio of the coverage area is α, and the weight ratio of the envelope volume is 1 − α, the following expressions are obtained:

(11) $$R_{W}=\alpha \times S_{new}+1-\alpha \times V_{new}\;,$$

where $R_{W}$ is the normalized weighted value of the coverage area and the envelope volume; $S_{new}$ denotes the dimensionless normalized coverage area; and $V_{new}$ stands for the dimensionless normalized envelope volume. Considering the coverage area and the envelope volume in terms of the geometric parameters, the weight of the network volume is equivalent; generally, α is taken as 0.5.

#### 2.2.2. R_E_ + V_E_

In rock mechanics, the instability of the main weight-bearing body and the failure of key structures are the results of a series of displacements and deformations that occur when active or passive energy accumulates and breaks through the steady-state critical value. Among these, a force such as deformation energy is an important scientific index in rock mechanics research. The main characteristics are as follows: the internal deformation of the rock mass structure can produce continuous accumulation, which means that the energy accumulates slowly. When the energy accumulation reaches the steady-state critical value, it will be released either actively or passively, causing damage to the rock mass structure, and thus affecting the engineering stability. This forces us to pay attention to the energy evolution characteristics at any particular time, from stress and strain to energy change, to deformation and displacement, which is also the ideal state that must be achieved by our MSM system. This study starts with energy and optimizes the grid layout scheme, based on the ability and sensitivity of the energy perception in the research grid layout system, such as the coverage area and the envelope volume mentioned above. 

To evaluate a network layout plan, maximizing the comprehensive monitoring capability of its network is the ultimate goal that needs to be continuously optimized and improved, considering the complexity of rock mass mechanics and its failure form, failure state, and energy transfer characterization methods and mathematical relationships. Based on the commonly used estimation indicators of source failure potential, energy and magnitude are widely used by the relevant scholars and scientific research institutions [34]. In this study, energy is used as the main monitoring and evaluation index parameter of the station network. Magnitude can be converted to energy, as follows:

(12) logE=c+dM 

where E stands for the source energy; M represents the source magnitude; d is usually 1.5; and c depends on the specific working conditions of the mine.

Specifically, the energy radiation radius ($R_{E}$) and the critical sensing-space envelope volume ($V_{E}$) of each level of energy in the sensor network are used as the calculation parameters. Among them, the detectable radius is closely related to many factors, such as the sensor’s attributes, the target monitoring frequency band, and the on-site environmental conditions. The farther the monitoring distance between the source and the equipment, the higher the induction degree of the lowest-energy events that can be monitored, and the better the monitoring performance. When the sensor equipment is selected, the on-site working condition is the key and most variable influencing factor. It is necessary to fully consider the source energy radiation range and attenuation distance, and then extract and analyze the data obtained from the on-site observation data of a specific mine, as this is the most real and effective way. From the literature [18,19,35,36,37,38], it has been established that the macroscopic mathematical problem is mainly reflected in the energy, *E*, of the microseismic event between the monitoring radius, *R*, and its derivative relationship:

(13) $$E=\mu \times R^{q}\;$$

where E represents the source energy; R denotes the monitoring radius; *q* is generally close to 2; and *μ* and *q* need to be statistically calculated in the corresponding mine data. 

**Figure 5:**
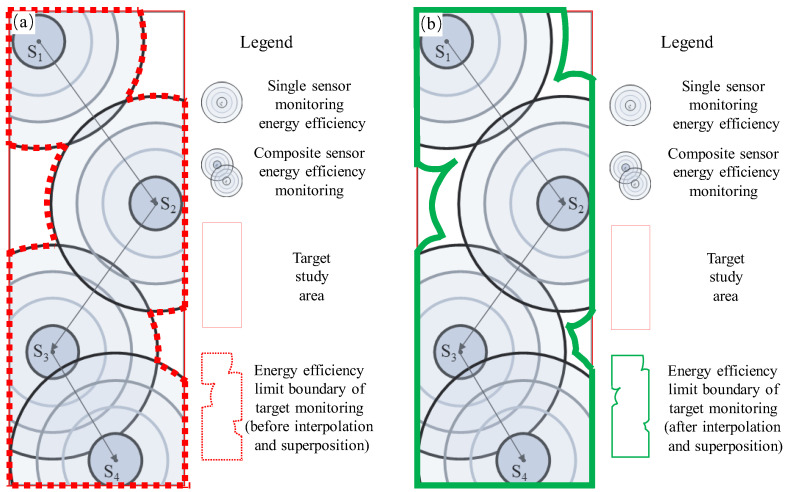
Schematic diagram of the minimum energy magnitude induction sensitivity envelope before and after interpolation superposition: (**a**) the schematic diagram of the energy efficiency range of a single sensor; (**b**) the schematic dia-gram of the superposition of the energy efficiency of the sensors under the composite condition.

The specific schematic is shown in Figure 5. Specifically, within a certain distance range, the ability to monitor the event’s energy level is derived from the detectable radius, which is obtained from the statistical data of the mine. According to the mine’s field data, the radiation energy of each sensor and its corresponding monitored events are analyzed, and the relationship between the energy attenuation and the radiation radius is comprehensively analyzed and fitted. In the network layout scheme, the maximum sensing radius of the smallest event that can be detected by each sensor is superimposed upon the others to form a new energy level sensitivity envelope, which is interpolated to obtain the lowest energy level sensitivity envelope and volume space parameters and, finally, calculate the new envelope volume. 

The maximum coverage radius, matched by the minimum magnitude energy obtained with Equation (13), is combined with the weighted combined advantages of the coverage area and the envelope volume. The envelope volume after the actual superposition is obtained. The formula is as follows:

(14) $$V_{E}=\int_{Z_{min}}^{Z_{max}}S_{XOY}dZ\;,$$

where $V_{E}$ is the envelope volume after the actual superposition of the maximum coverage radius, matched by the minimum magnitude energy; and $S_{XOY}$ represents each of the spatial bodies formed by the sensor array in the Cartesian coordinate system, differentiated in the plumb direction. In the geometric area of the XOY surface, dZ is the differential in the Z direction, $Z_{min}$ denotes the lower limit of the geometric space elevation formed by the sensor array, and $Z_{max}$ represents the upper limit of the geometric space elevation formed by the sensor array. 

### 2.3. Formula Optimization

Referring to the literature [19], based on Equation (6) and the above analysis key indicators (S, V, $R_{E}$, and $V_{E}$), the following improved calculation methods and corresponding formulas are constructed, as follows:

(15) $$C_{min}=\int_{R_{min}}^{R_{max}}\int_{Z_{min}}^{Z_{max}}R_{W}\times S_{XOY}\times E_{R}\left(R\right)\times f\left(C_{x}\right)dZdR,s\in \varphi ,\;R\in R_{a}\;$$

where $C_{min}$ is the final evaluation value for the optimal configuration standard of the stations in the MSM network; $R_{min}$ and $R_{max}$ are the respective maximum propagation radii of each sensor at different energy levels; $R_{a}$ represents the collection of sensors in the network ($R_{a}=\left[R_{1},R_{2},\ldots ,R_{n-1},R_{n}\right]$); $Z_{min}\;\mathrm{and}\;Z_{max}$ are the same as the items in Equation (14); $R_{W}$ is Equation (11); and $E_{R}\left(R\right)$ denotes the internal network. The magnitude energy value corresponding to each sensor in the different radius ranges is the range that the station network can accept, in terms of the different energy levels of the source-induced events. $C_{x}$ is the covariance matrix of x; the constants and variables of the f function depend on the physical properties of the problem and meaning. dR represents the differential of the radius, R, of each sensor with different energy levels, s denotes the set of station coordinates in the network, and φ is the spatial domain of potential station locations. 

The optimal estimation method for the station network, based on the coordinates of the stations and the source coordinates in the MSM network, is the most practical optimization method. On the one hand, when calculating the covariance matrix, C, based on the D-value optimization theory, the arrival time error, the uncertainty of the velocity model, and the variance of the source event parameters are considered. On the other hand, thanks to the D-value optimization method, which relies upon a classical theoretical basis and the scalability of its unknown parameters, the attribute assignment and positive correlation of the key parameters are increased in the traditional covariance confidence ellipsoid evaluation. Based on this relationship and the abovementioned deduction formula, the configuration of the network layout scheme is verified by the numerical simulation method. According to the calculation result map of the S-V-E-R-V model, a comprehensive evaluation and display of the configuration quality of the MSM network can be carried out.

### 2.4. Solution Process

Considering the problem of identifying the key parameters of the constructed S-V-E-R-V model, the controllability of the sensor layout variables of each scheme needs to be consistent. Among them, clear sensor placement information is the key information needed to obtain the coverage area and establish the envelope volume parameters. In addition, the radiation radius and energy level sensitivity are the main parameters of the calculation and comparison process. This model can quickly determine and compare the optimal layout plan, based on the above key information. 

Step 1: Build a sensor coordinate selection library. First, we delineate the range in each middle section of the research space, particularly to meet the monitoring needs of the main ore body range and the main structures and facilities. Second, we select the proposed sensor points in all suitable locations, such as roadways and stopes, where sensors may be installed. Third, we synthesize all the proposed points to calculate and analyze the spatial boundary of the model and build the model. Fourth, we derive the three-dimensional spatial coordinates of all the proposed sensor points.

Step 2: Select a combination scheme. Select the coordinates of a certain number of sensors from the coordinate library described in step 1 and select them in sequence according to the combination method in mathematics to form the first, second, third, …, *n* − 1, and *n* types of combination schemes.

Step 3: Calculate the key parameters in the S-V-E-R-V model. First, concerning the above analysis and transformation equations (see Equations (7)–(12)), we calculate the overall global coverage area and the coverage area of each target middle section under different radiation radius conditions. Second, according to the existing data and Equation (13), we conduct statistical analysis and fitting of the specific relationship between the radiation radius and the energy level. Third, we determine the volume enveloped by the energy levels of each level under the condition of different radiation radii.

Step 4: Weighted comparison of key parameter data, such as the overall coverage area under different radiation radius conditions (coverage area in different projection directions), the coverage area of each middle section with different radiation energy levels, and the envelope volume of each radiation radius.

Step 5: Repeat steps 3 and 4 in sequence, according to the combination scheme of step 2 and its sequence, until the best result is obtained.

Step 6: Determine the plan, produce the resulting diagram, and provide engineering guidance. 

Due to the large volume of data from the proposed points database and the proposed coordinates, the number of all possible combinations is large, and a certain amount of thinning retrieval is required. On one hand, the data redundancy and combination scheme redundancy caused by a large number of calculations is excluded; on the other hand, it is more in line with the density of the sensor points to be selected in an actual project layout and the ore mining construction technology. In the solution process, the combination scheme of each group of sensors will have the calculation and storage of the key parameters in this model, which is convenient for making a weighted comparison with the sequence scheme until the optimal result is obtained. 

## 3. Case Analysis

**Figure 6:**
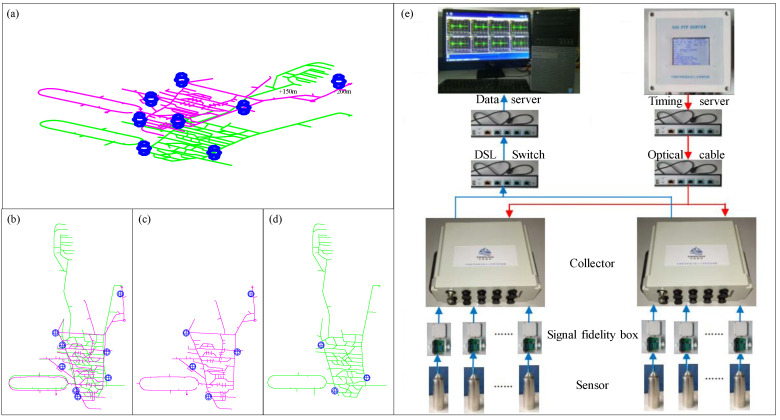
Mine development layout and the existing MSM sensor network layout engineering drawing: (**a**) 3D perspective view; (**b**) overall top view; (**c**) +200 m middle section plan; (**d**) +150 m middle section plan; (**e**) hardware equipment connection diagram.

The feasibility of this model method is demonstrated by the following cases. The second-phase MSM system of the Xinjiang Ashele Copper Mine is used as an example for analysis. The mine has a development layout within an MSM sensor network layout project. Several sensors are mainly arranged in the +150 m middle section and the +200 m middle section, respectively. The specific global three-dimensional view, global top view, and the positional relationship of each middle section are shown in Figure 6a–d. To determine the specific coordinates of the fracture sources and sensors in the mine rock structure, a Cartesian coordinate system has been established. Then, with the help of a full set of Zhongke MSM System (http://www.sinoseism.com/, accessed on 30 May 2022) hardware equipment, tests on monitoring and positioning, data processing, and analysis were carried out. The physical structure of the hardware equipment connection is shown in Figure 6e. 

The location of the sensor and its ancillary properties have a great influence on the source location results. Against this engineering backdrop, in order to verify the practicability of the above optimization method, according to the deployed sensor station network, its actual monitoring effect was analyzed, and a comparison basis for the optimized layout scheme was conducted. The specific coordinates of the eight sensor coordinate points in the original layout plan shown in Figure 6 are given in Table 2.

**Figure 7:**
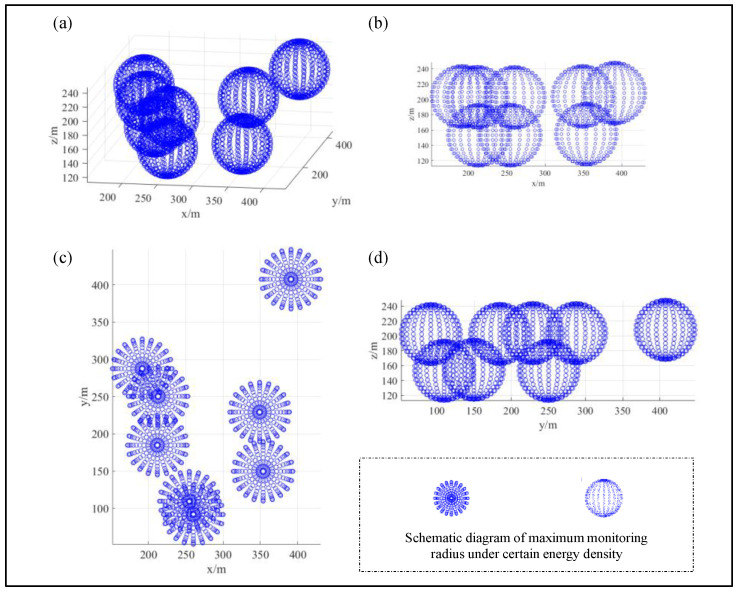
Analysis chart of the monitoring efﬁciency of the mine’s current network layout: (**a**) Global stereo view; (**b**) XOZ normal top view; (**c**) XOY normal top view; (**d**) YOZ normal top view.

To use this method to evaluate the existing sensor network in Phase II of the Ashele Copper Mine, it was first necessary to calculate the coverage area and the envelope volume of the network, composed of all sensors (as shown in Figure 7). Second, the minimum coverage radius of each sensor in the previous monitoring data was analyzed, and the monitoring range of each energy level of each sensor was drawn up (as shown in Figure 5a); that is, its sensitivity index and distribution, and the monitoring energy levels in different coverage radii were superimposed (as shown in Figure 5b). Finally, numerical interpolation and superposition calculations were carried out on the monitoring ranges of the different energy levels of all sensors, and the actual monitoring optimum coverage volume for optimal performance was obtained. After data statistics analysis, it was found that in the second phase of the mine site project, the average monitoring radius of each sensor during the 24-month monitoring period in 2018 and 2019 was 165.8 m. Taking the minimum monitoring radius, R (50 m), from the statistical results as the assumption analysis, the monitoring radius corresponding to the energy range is shown in Figure 7. 

In the S-V-E-R-V model, the monitoring efficiency of the network layout is determined by the maximum coverage radius and the envelope volume after the superposition of all interpolations under the condition of the lowest monitoring energy level. To accurately calculate the relationship between the actual propagation radius and the monitoring energy level, in addition to consulting the relevant literature, it is more important to combine the long-term effective monitoring data from actual mining engineering to calculate a mathematical relationship that conforms to the actual working conditions. Based on Equation (13) and the 24-month effective mine-monitoring data, the relationship with the energy E monitoring radius R of the microseismic event, in this case, can be expressed as:

(16) $$E=3.748\times 10^{-4}\times R^{3.0810}\;.$$

**Figure 8:**
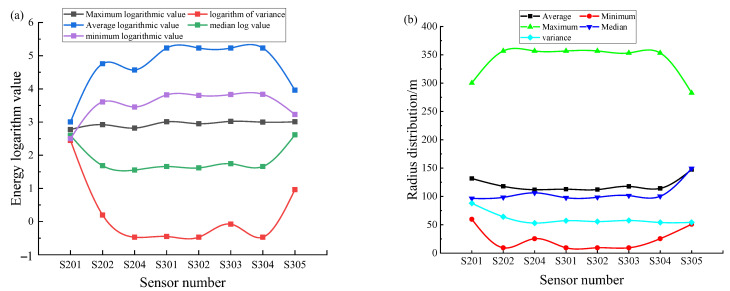
Research target sensor monitoring the energy range and radius distribution map: (**a**) distribution of energy logarithm corresponding to each sensor; (**b**) distribution of radiation radius value corresponding to each sensor.

Specifically, this analysis is based on the MSM data of the +150 m middle section and the +200 m middle section of the second phase of the mine. The intuitive distribution of the statistical data values is shown in Figure 8. Figure 8a shows the intuitive distribution relationship between the sensors in the target study middle section and their log energy values, and Figure 8b shows the intuitive distribution relationship between the sensors in the target study middle section and all sensing radius values in the statistical period. 

**Figure 9:**
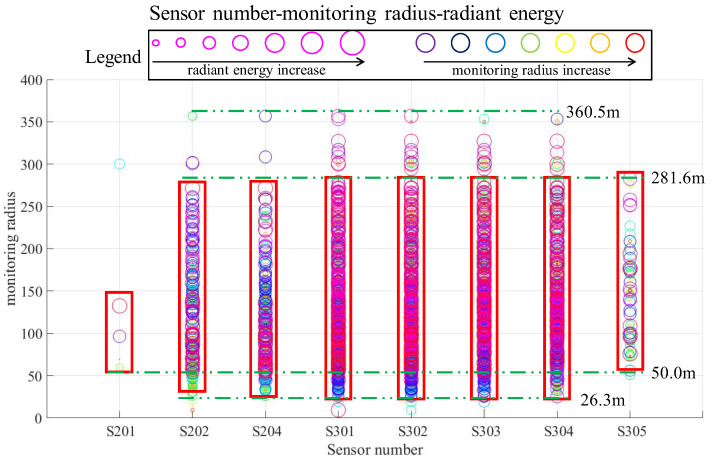
Summary of the monitoring radius and energy distribution corresponding to sensors in the study area.

Figure 9 shows the distribution of all sensing radii and the energy size of each sensor in the statistical period of each sensor in the current research area of the mine’s MSM system. The use of small to large circles in the figure indicates that the monitoring radiu is from small to large, and the use of color from cool to warm in the figure indicates that the event energy is from small to large. Except for those individual values that are extremely large or extremely small, the minimum monitoring sensing radius is usually 26.3 m, and there are also a few cases where it is 50.0 m. The maximum value is 360.5 m, but the proportion of events is small, and these events with a large radiation radius and high energy level are more often at 281.6 m. On the one hand, the relationship between the energy of the microseismic event and the monitoring radius conforms to the mathematical relationship expression deduced above and, therefore, can be statistically analyzed and verified at the mine site; on the other hand, the statistical results provide a hierarchical research basis for the subsequent model-building process. The choice of different radiation radii has a reference basis, along with analytical and comparison significance. 

**Figure 10:**
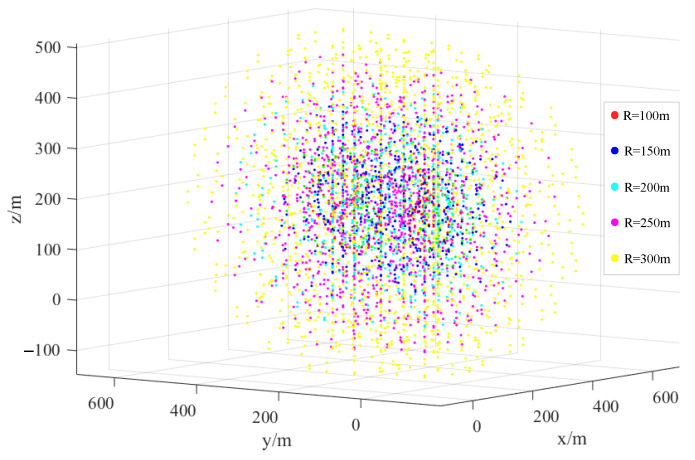
Distribution of the independent point clouds of each sensor according to the different monitoring energy levels.

Referring to the existing statistical data of the mine and the analysis of the assumption of the minimum radius and Equation (13), the data of the proposed five levels of the radius (100 m, 150 m, 200 m, 250 m, and 300 m) correspond to the monitoring energy sensitivity, and the sensitivity is assigned. To distinguish the monitoring range of different energy levels of independent sensors and their comprehensive monitoring range after all sensors are superimposed, the distribution of the independent point clouds of each sensor in different monitoring energy levels is shown in Figure 10. Specifically, it is a schematic diagram to distinguish the paving steps of determining the coverage area and the envelope volume for interpolation calculation and, especially, to lay the foundation for the calculation of the envelope volume of the energy-sensing sensitivities corresponding to the different monitoring radii of all sensors.

**Figure 11:**
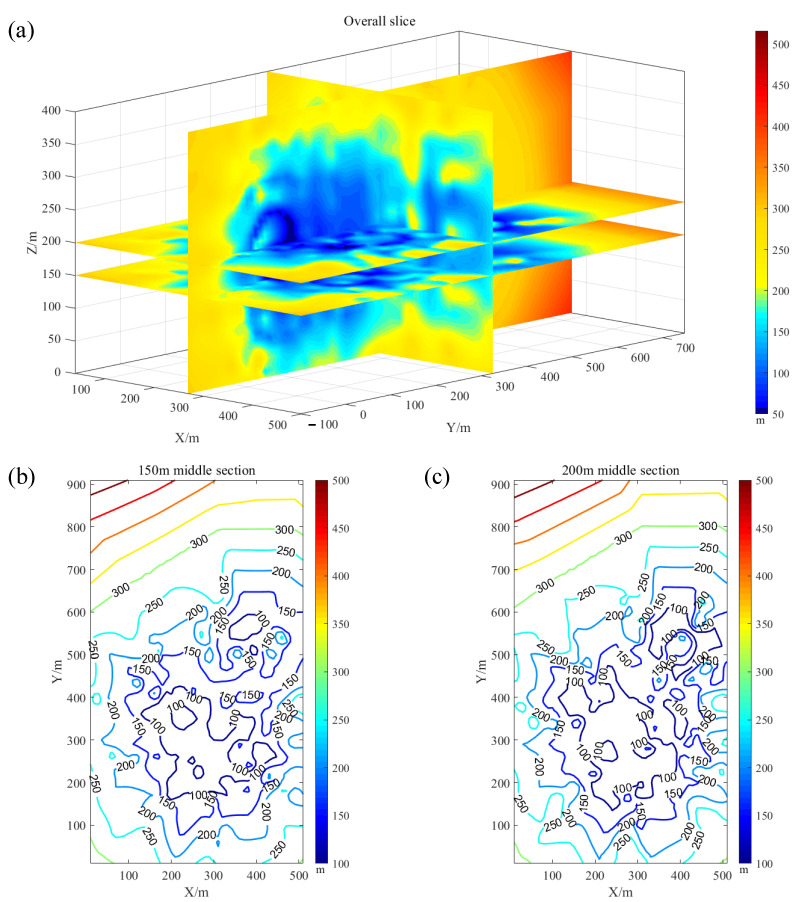
The result of the interpolation calculation of the current deployment’s network efficiency: (**a**) overall slice; (**b**) 150 m middle section; (**c**) 200 m middle section.

From the basic schematic diagram and pavement of Figure 7 and Figure 10, we can easily assume that the monitoring efficiency of the actual network in the mine MSM system is based on the maximum envelope range formed by the mutual superposition of various sensors. At this point, the coverage area, envelope volume, and target energy level corresponding to the maximum monitoring volume enclosed within the radiation radius yield the most accurate basic reference data, which can be calculated and compared with the compound covariance in Equation (15). Verification and the comparative basic data for the subsequent optimization of the method can also be provided. First, under the condition that eight sensors are deployed at the same time and are effective, the interpolated cloud map of the different energy level coverage of the study area (as shown in Figure 11a), and the interpolated contour map of the radiation radius in the middle section where the sensors are deployed, can be obtained (see Figure 11b,c). 

From the display in Figure 11 and the analytical process of the aforementioned formula, it can be seen that it is helpful to observe the monitoring sensitivity of the energy levels of monitoring events in different regions and to make visual sensory judgments to carry out the primary optimization of the network layout method. This procedure is used to obtain the radiation radius data and calculate the required geometric parameters, to provide detailed data support for the comparability and applicability of the optimization scheme. Then, the most important thing is to help judge the different distributions of sensors in the network and the different monitoring effects. In addition, its visualization and data characteristics are obvious, which is convenient for improving the efficiency of network optimization and analysis. Specifically, the higher the relative independence of a single sensor, the worse its peripheral monitoring performance, and the lower the energy level-sensing sensitivity. The more inclusive the multiple sensors, the better the monitoring performance within a specific range, and the energy level sensing sensitivity in general. When the monitoring range is fixed, the farther away from the sensor, the worse the monitoring efficiency, and the lower the energy level-sensing sensitivity. 

**Figure 12:**
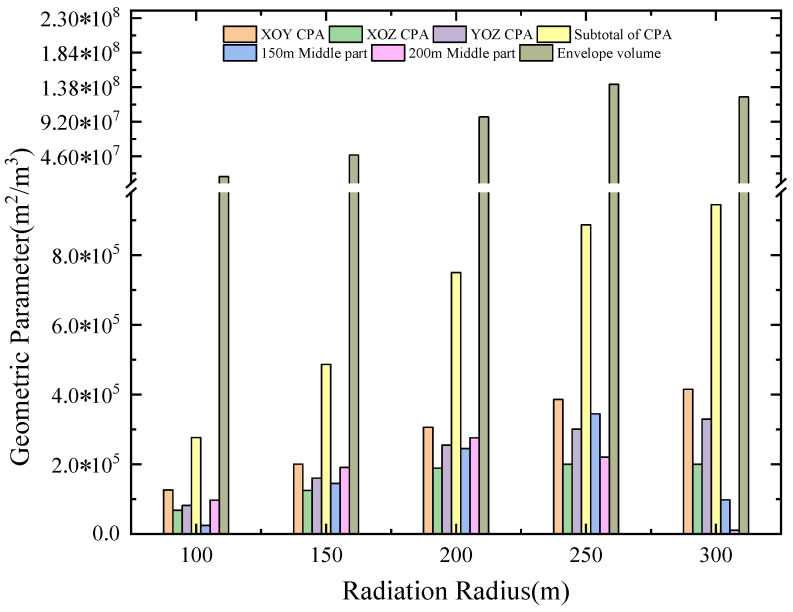
Evaluation results of the new method for the current grid layout of the mine.

In addition to comparing and analyzing the point-cloud maps of the abovementioned network with different radiation radii and the slice maps of the whole and of each middle section, it is important to calculate the corresponding monitoring range data. The evaluation criteria and data results in the actual analysis of the existing engineering case are used as the basic comparison data source for subsequent method optimization. The overall coverage area is calculated, based on Equation (7) and Figure 11 above, according to the different radiation radius levels, and the global projection area maximization joint analysis and its calculation. The coverage area parameter value under this deployment situation (the coverage area of different radiation radius levels in each middle section) is more specific and true to the sensor network deployment quality of the target middle section, which is the key parameter of the coverage area index. The maximization of the envelope volume of the different levels of radiation radii reflects the overall monitoring efficiency of the network layout, especially since, once the radiation radii and the corresponding minimum energy level sensitivity are converted, the practical significance of this parameter is more relevant. The specific main research parameters of the existing second-phase project network layout are the overall coverage radius of each projected area and its mean value, the coverage area of each middle section with the different radiation radii, and the envelope volume of the radiation radius at all levels (for detailed parameters, see Table 3). A specific comparison of the current layout plan of the mine is shown in Figure 12.

## 4. Numerical Simulation Analysis

**Figure 13:**
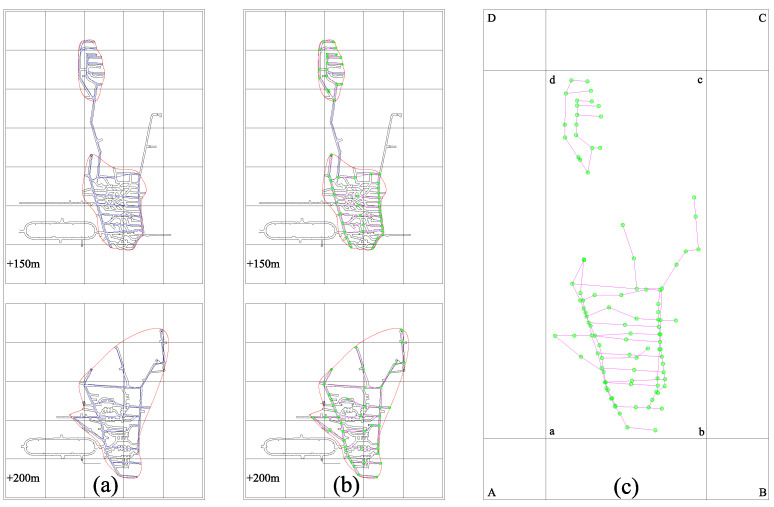
Model construction of the network layout in the target area of the mine: (**a**) delineation of the monitoring range; (**b**) proposed sensor points; (**c**) construction of a simulation model and boundaries: a, b, c, d are the actual boundary limit coordinate point considering the sensor point database; A, B, C, D mean boundary limit coordinate points for numerical simulation considering sensor monitoring efficiency.

Due to the different placement positions of sensors in the network, the geometric structure formed by the layout of a particular network and its energy-sensing sensitivity determine the comprehensive monitoring efficiency of the network. The abovementioned method, based on an analysis of the S-V-E-R-V model for the key parameters of the existing sensors in the mine, is only used as the basic comparison data source for the subsequent program optimization. To further verify and promote the application of this method, the solution steps of the new method can be followed and numerical simulation can be used to analyze the possible potential grid layout forms of the mine, one by one, to further compare and optimize the optimal grid layout scheme. Here, to evaluate the monitoring efficiency of the network layout in the monitoring area, all possible sensor locations are identified from the middle of the second phase of the mine project to form a sensor coordinate database and to build a numerical simulation model and its boundaries (as shown in Figure 13). 

The basic principle of selecting sensors is as follows: according to the proposed coordinate database, it is necessary to delineate potential sensor points and record the data. There are 99 proposed coordinate positions in the two middle sections, including 55 in the +150 m middle section and 44 in the +200 m middle section. To study the maximum monitoring efficiency of a certain network layout, the sensor coordinate database is combined, referring to the statistical data of the mine and the above analysis to comprehensively select different radii of 100 m, 150 m, 200 m, 250 m, and 300 m, and to calculate the radiation. For the energy level, we select the maximum monitoring range that corresponds to the lowest energy level that can be sensed by the grid and compare the geometric parameters of the solver horizontally to obtain the best layout plan. 

**Figure 14:**
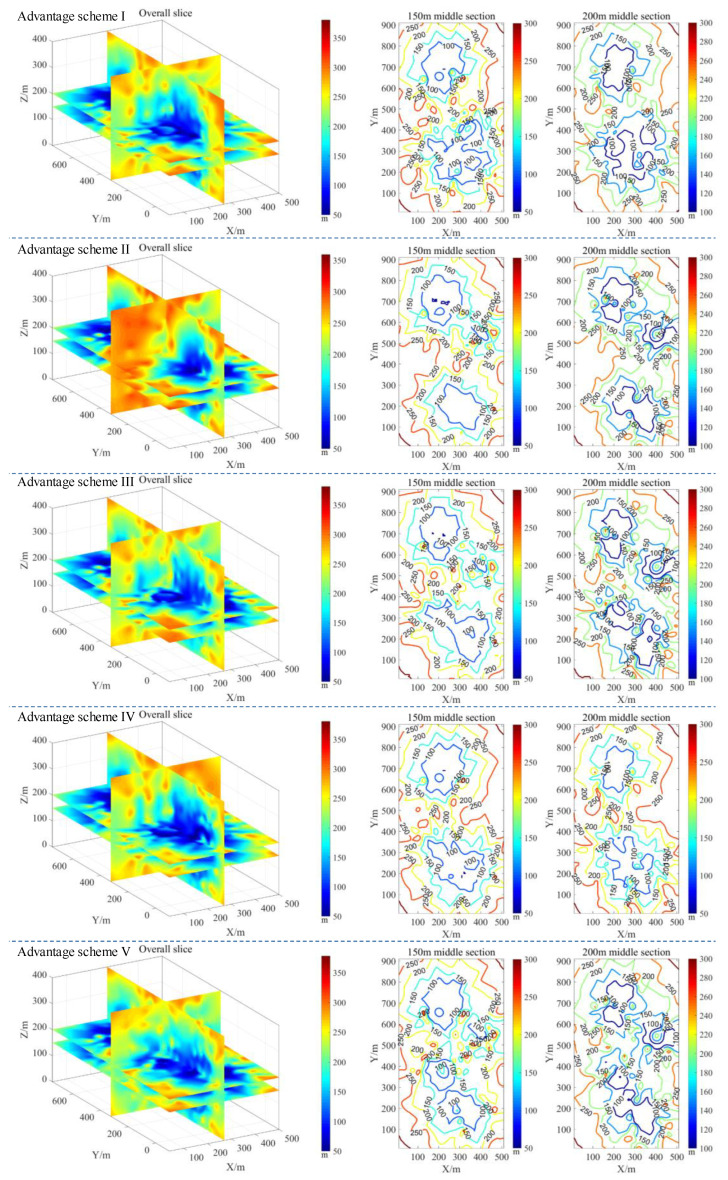
Optimization results of the network layout: monitoring efficiency in the case of different middle sections and different radiation radius conditions.

Figure 14 shows the monitoring efficiency results of the five optimal networks, determined using the above optimization method at different radiation radius energy levels of 100 m, 150 m, 200 m, 250 m, and 300 m. The color change from cool to warm indicates that the minimum energy level required to monitor the event is becoming increasingly higher. In other words, if you need to achieve a larger monitoring range performance, you need a certain mesh layout to sense events that are more distant using smaller energy levels. By comparing the envelope ranges of the monitoring energy level sensitivity under different radiation radii, and then performing a weighted comparison, five optimal layout schemes are selected. This is in sharp contrast to the current monitoring network used in mines, which is established via the monitoring range of different energy levels. In addition, the network layout optimized using the new method provides a more obvious data comparison under different radiation radii, indicating that the new method is superior to the traditional method. 

**Figure 15:**
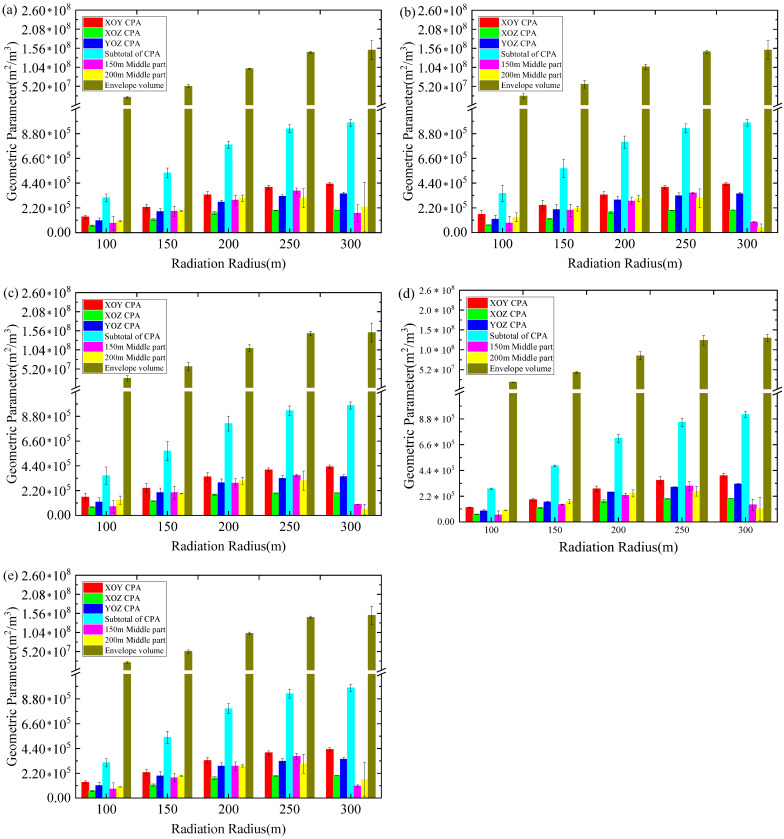
Average absolute distance error and standard deviation of the calculation results of various geometric parameters under multi-level radiation radii: (**a**) advantage scheme Ⅰ; (**b**) advantage scheme Ⅱ; (**c**) advantage scheme Ⅲ; (**d**) advantage scheme Ⅳ; (**e**) advantage scheme Ⅴ.

Under the conditions of different levels of radiation radius, as the radiation radius increases, the geometric parameters adapt to the increase and the corresponding monitoring performance of the network is optimized, based on meeting the needs of the mine, which verifies that the use of the S-V-E-R-V model has obvious advantages. Figure 15 shows the calculation results of the comparison between the first five optimal schemes after optimization and the mean absolute error and standard deviation of the mine’s existing grid layout. Since the effective coverage range of the 300 m radiation radius in all optimization schemes is not fully covered in this figure, when assessing the calculation effectiveness of the minimum monitoring energy level, the geometric parameters covered by the radius of 250 m and below are primarily considered. It can be seen from Figure 15 that the five optimal schemes, optimized based on this model, all have more advantageous analysis results than those of the current mine layout. It can be seen that the S-V-E-R-V model is helpful for determining the layout of the mine MSM network and for the optimization of the scheme and that the room for error is small, indicating that the method is highly appropriate. Therefore, this method is suitable for the comparison and optimization of existing MSM network deployment systems in mine engineering, and has high applicability, especially for mines that have never employed an MSM network. 

## 5. Discussion and Conclusions

This paper proposes a method for optimizing the mesh distribution of microseismic monitoring stations in mines and other underground mine engineering scenarios, based on the S-V-E-R-V model. The advantages of this method are as follows: (1) Being aimed at monitoring efficiency, it maximizes the monitoring range, monitoring mobility, and field transition efficiency, thus meeting actual engineering needs; (2) The S-V-E-R-V model and its indicators are easy for front-line technicians to understand, and they can quickly obtain and build a practical model that includes the coverage area of the network (S), the envelope volume of the network (V), the coverage radius of each energy level of the network (RE), and the effective energy level sensitivity monitoring envelope volume (VE), so as to comprehensively evaluate and optimize the monitoring performance of the network layout; (3) With regard to visualization, the expected monitoring energy level sensitivity in the target monitoring area can be dynamically visualized in real time using this method, thus providing a data comparison and analysis basis for the judgment of various schemes, making the results more intuitive and improving their credibility. The accuracy of the method is verified by the simulation comparison between this method and the existing scheme in the mine site. The results show that this method has better monitoring efficiency and practicability than the traditional methods. Internal iteration and optimization are carried out in this method, and the simulation results show that it is always possible to optimize the optimal network layout plan by an iterative cycle, with a strong self-correction ability.

However, this method has the following limitations: (1) the theoretical basis of the network layout is relatively weak; (2) the innovation of the network layout and the adaptability of the engineering site still need to be optimized; (3) the promotion of all underground mine application platforms is difficult; and (4) the demand for fast, efficient, and intelligent mining sites is the development direction of the network layout. Therefore, a network layout optimization method that is capable of automatic iterative optimization and deep learning is of great significance and deserves further research.

## Figures and Tables

**Table 1:** Application effects and limitations of the current typical network optimization methods.

No.	I	II
Classification name	Basic mathematical and physics theories and algorithms	Combined with a theoretical basis and engineering practices
Method summary	Monte Carlo method [14] D-value optimization algorithm and its improvement method [15,16,17,18,19,20,21] C-value optimal design theory and its improvement method [22] Genetic algorithm [23] Machine learning and deep analysis [24,25,26]	Numerical analysis method [27] Wave speed correction method Engineering location and primary and secondary zoning method [28] Energy decay method Software-corrected inversion method [29] Comprehensive evaluation method [30]
Features introduction	Advantages: Realize the local and regional optimization of the network layout, and successfully optimize the positioning efficiency of the engineering site.	
	Disadvantages: 1. The actual needs of the engineering site determine the sensor deployment area in the network: the existing theoretical optimization methods are limited to theoretical assumption models and calculations at the numerical level, and there is a certain gap in terms of practicability in line with mine engineering sites. 2. The specific quantitative indicators for maximizing the monitoring range are not clear: under the actual conditions of the project site, the actual measurement indicators and evaluation systems are quite different. This needs to be combined with the actual coverage area and envelope volume of the network layout and its monitoring and sensing sensitivity. 3. Continuous changes in different dynamic engineering cycles require more rapid and stylized optimization methods.	

**Table 2:** Mine target analysis area sensor installation coordinates.

Serial Number	1	2	3	4	5	6	7	8
Numbering	S150-6	S150-7 *	S150-9	S200-10 *	S200-11	S200-12	S200-13	S200-14
x (m)	254.71	212.83	353.7	260.09	211.8	191.53	349	391.01
y (m)	109.51	250.27	149.6	91.61	184.64	287.44	229.1	407.55
z (m)	153.2	153.1	154.6	202.5	202.7	203.7	203.8	208.2

Note: (1) The coordinates in the table have been transformed without changing their relative positional relationship; (2) the coordinates with ‘*’ are three-way speed sensors, and the rest are one-way speed sensors.

**Table 3:** Summary of main research parameters of the existing second-phase project grid layout.

Radiation Radius/m	Overall Coverage Area/m^2^ Projection Plane				Coverage Area of Each Middle Section/m^2^		Radiation Radius Envelope at All Levels/m^3^
	XOY	XOZ	YOZ	Subtotal	150 m	200 m	
100	1.27 ×10^5^	6.85 × 10^4^	8.18 × 10^4^	9.23 × 10^4^	2.48 × 10^4^	9.71 × 10^4^	1.89 × 10^7^
150	2.01 × 10^5^	1.25 × 10^5^	1.61 × 10^5^	1.62 × 10^5^	1.45 × 10^5^	1.91 × 10^5^	4.76 × 10^7^
200	3.06 × 10^5^	1.89 × 10^5^	2.55 × 10^5^	2.50 × 10^5^	2.45 × 10^5^	2.76 × 10^5^	9.83 × 10^7^
250	3.86 × 10^5^	2.00 × 10^5^	3.01 × 10^5^	2.96 × 10^5^	3.45 × 10^5^	2.21 × 10^5^	1.42 × 10^8^
300 #	4.15 × 10^5^	2.00 × 10^5^	3.29 × 10^5^	3.15 × 10^5^	9.80 × 10^4^	1.06 × 10^4^	1.25 × 10^8^

Note: # indicates that the data has no actual envelope in some areas within the research range.

## Data Availability

The data presented in this study is available on request from the corresponding author.
